# Influence of new societal factors on neovascular age-related macular degeneration outcomes

**DOI:** 10.1186/s12886-018-0690-9

**Published:** 2018-02-01

**Authors:** Audrey Giocanti-Aurégan, Elige Chbat, Adil Darugar, Christophe Morel, Bruno Morin, John Conrath, François Devin

**Affiliations:** 1 Department Ophthalmology, Avicenne Hospital, DHU Vision and Handicaps, 125 rue de Stalingrad, 93000 Bobigny, France; 2 Sorbonne Universités, UPMC Univ Paris 06, INSERM, CNRS, Institut de la Vision, 17 rue Moreau, 75012 Paris, France; 3 Ophthalmology Center Paradis Marseille, 433 rue Paradis, 13008 Marseille, France; 4 Pitié Salpêtrière Hospital, Ophthalmology, DHU Vision and Handicaps, 47-83 Bd de l’Hôpital, 75013 Paris, France; 5 Oise Ophthalmology, 6 Avenue du Poteau, 60300 Chamant, France

**Keywords:** Age-related macular degeneration, Economic burden, Societal factors, Retinal disease

## Abstract

**Background:**

To assess the impact of unstudied societal factors for neovascular age-related macular degeneration (nAMD) on functional outcomes after anti-VEGFs.

**Methods:**

Charts of 94 nAMD patients treated in the Monticelli-Paradis Centre, Marseille, France, were reviewed. Phone interviews were conducted to assess societal factors, including transportation, living status, daily reading and social security scheme (SSS). Primary outcome was the impact of family support and disease burden on functional improvement in nAMD.

**Results:**

Between baseline and month 24 (M24), 42.4% of the variability in best-corrected visual acuity (BCVA) was explained by the cumulative effect of the following societal factors: intermittent out-patient follow-up, marital status, daily reading, transportation type, commuting time. No isolated societal factor significantly correlated with ETDRS BCVA severity at M24. A trend to correlation was observed between the EDTRS score at M24 and the SSS (*P* = 0.076), economic burden (*P* = 0.075), time between diagnosis and treatment initiation (*P* = 0.070). A significant correlation was found for the disease burdensome on the patient (*P* = 0.034) and low vision rehabilitation (*P* = 0.014).

**Conclusions:**

Societal factors could influence functional outcomes in nAMD patients treated with anti-VEGFs. They could contribute to the healing process or sustain disease progression.

**Electronic supplementary material:**

The online version of this article (10.1186/s12886-018-0690-9) contains supplementary material, which is available to authorized users.

## Background

Age-related macular degeneration (AMD) is the leading cause of blindness in developed countries [1–5] and is responsible for 8.7% of all blindness worldwide. AMD prevalence is expected to increase with population ageing projections [6]. AMD is a multifactorial and heterogeneous disease and many studies have investigated the effects of environmental factors such as education, socioeconomic status [7], smoking [8, 9], alcohol consumption [10], cardiovascular diseases [11], diet, obesity, oestrogen levels, light exposure, statin and aspirin therapies [12] on AMD development and progression.

In a recent study assessing twins, Seddon et al. [13] have suggested that both genetic and environmental proportions of variance were observed for specific macular drusen and retinal pigment epithelial features. In addition, Keilhauer et al. [14] have also suggested that despite genetic risk variants predisposing to AMD, the course and visual outcomes appear to be affected by environmental factors rather than genetic determinants. At the time of our study, neovascular AMD (nAMD) treatment included a monthly monitoring and/or injections requiring a scheduling for patients and caregivers, and resulting in a significant burden on patients and their family. This therapeutic scheme involves new societal factors that may influence the course and functional outcomes of nAMD patients treated with anti-VEGFs.

While numerous studies agree on the effect of the above-mentioned environmental factors, our study investigated the influence of new societal factors that are mainly related to anti-VEGF therapy. The aim of this study was to assess the impact of these new anti-VEGF-related societal factors on functional outcomes in nAMD patients.

## Methods

### Setting

The study was conducted in a tertiary care centre specialised in diagnosis and treatment of macular diseases, located in Marseille, France.

### Study design

The study was a retrospective analysis of patient charts treated for nAMD in the Monticelli Clinic with anti-VEGF intravitreal injections between October 2010 and October 2012 in a real-life setting. The societal data were obtained by phone interview. This study was conducted in accordance with the tenets of the Declaration of Helsinki, and an informed consent was obtained from subjects. Approval was obtained from the France Macula Federation ethical committee.

### Eligibility criteria

Consecutive patients diagnosed with nAMD, treated with anti-VEGF (ranibizumab) before October 2010 with a follow-up in the Monticelli Clinic of at least 2 years between October 2010 and October 2012, were included in this analysis. Patients who were not injected exclusively in the Monticelli Clinic and patients with other ocular disease responsible for vision loss were excluded.

### Methods

All patients were initially examined by an independent technician using an Early Treatment Diabetic Retinopathy Study (ETDRS) chart at baseline (October 2010) and their best-corrected visual acuity (BCVA) was regularly measured for 2 years. The visual outcomes assessed were baseline and final BCVA (M24) and change in BCVA during the study. The number of intravitreal injections received during this time was recorded. Another independent technician conducted phone interviews to assess parameters of interest: intermittent outpatient follow-up (yes/no), living status (in couple, with family or alone), type of vehicle used to come to the Monticelli Centre (taxi, public transportation, own vehicle, ambulance), commuting time (0–1 h; 1–2 h; more than 2 h); low vision rehabilitation (yes/no); daily reading type (journal, book, computer, none); healthcare system covering all expenses (yes/no); economic benefits of social security scheme (10-point scale) and the burdensome of the consultation for the patient (0–10-point analogic scale). We also collected patient complaints about their follow-up and treatment.

### Treatment regimen

All patients received ranibizumab injections at the Monticelli centre. After 3 loading-dose injections, patients were treated on an as needed basis. The as-needed treatment was administered if the patient had any of the following criteria in the study eye: loss of 5 or more letters of BCVA; new, recurrent or persistent subretinal or intraretinal fluid based on OCT; or new macular hemorrhage. The patients were monitored monthly by BCVA on ETDRS chart, retinography and time domain OCT.

### Data analysis

All statistical analyses were performed using XLSTAT version 2015. A Chi square analysis or Fisher’s exact test was used to assess the relationship between the BCVA score at M24 or the change in BCVA (on the ETDRS scale) over 24 months and all the above-mentioned societal factors. For the ANCOVA/multivariate unconditional logistic regression analysis, patients were divided into 2 independent groups: patients whose BCVA score evolved positively, and patients whose BCVA evolved negatively. *P*-values at 95% confidence interval [CI] for the chi square analysis or Fisher’s exact test were examined, as well as the coefficient of determination R^2^, analysis of covariance, model parameters, standardised coefficients at lower and upper limits (95%) using an ANCOVA analysis, for each demographic variable related to the BCVA score and evolution. Number of injections, age and baseline BCVA were controlled in the ANCOVA analysis.

The primary endpoint was to assess the impact of all the above-mentioned new societal factors on the BCVA at M24 and change in BCVA over 24 months.

## Results

Ninety-four patients with a mean age (±SD) of 77 (±8.42) years were included in the study. The mean BCVA at baseline was of 58.35 ± 13.65 letters, the mean variation over 2 years was of − 7.5 ± 10 letters (Min: − 58; Max: + 17), and the mean final BCVA was of 50.85 ± 11.8 letters. Patients have received a mean of 8.84 ± 3.9 (Min = 1; max = 21) injections before inclusion in the study, and received 6.74 ± 3.9 (Min = 0; max = 22) injections during the 2-year follow-up. There was no statistically significant association between the severity of the disease, measured with the final ETDRS score at M24 (October 2012) and the following demographic variables: intermittent outpatient follow-up (*P* = 0.821), family status (*P* = 0.564), daily reading (*P* = 0.180), commuting time to the clinic (0.782) and transport type (*P* = 0.743). However, a trend to dependence was observed between the BCVA at M24, and social security scheme (*P* = 0.083), and economic burden of the treatment (*P* = 0.075). Low vision rehabilitation (*P* = 0.014) and burdensome of the consultation for the patient (*P* = 0.034), significantly correlated with BCVA severity at M24. Also, the time between the diagnosis and the initiation of the treatment tended to correlate with the BCVA score at M24 (*P* = 0.070). The injection number during the 2-year follow-up was not related to BCVA severity at M24 (*P* = 0.999).

**Fig. 1:**
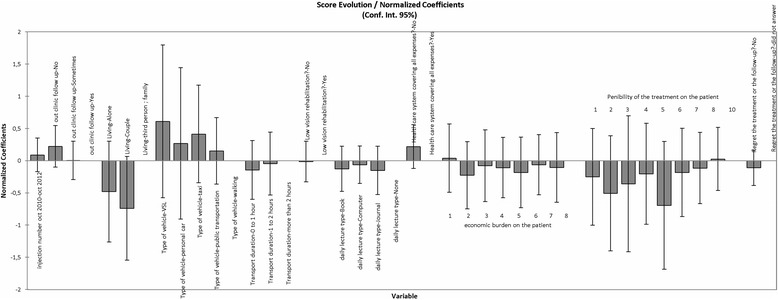
Graphical representation of the model assessing the weight (coefficient) of each variable on the change in ETRDS score over 24 months

Regarding the link between each demographic factor and the change in ETDRS score over 2 years (between baseline and M24), no significant correlation was observed with: intermittent outpatient follow-up (*P* = 0.943), family status (*P* = 0.308), daily reading (*P* = 0.752), commuting time to the clinic (*P* = 0.175), transport type (*P* = 0.157), security social scheme (*P* = 0.129), score of the consultation burdensome for the patient (*P* = 0.468), economic burden of the treatment (*P* = 0.654), and low vision rehabilitation (*P* = 0.176). The number of injections required was not significantly related to the change in ETDRS between baseline and M24 (*P* = 0.128). The distribution of the different variables is summarized in Table 1 (and Additional file 1) for patients with a negative evolution of their ETDRS score over the 24-month follow-up, and in Table 2 (and Additional file 1) for those with a positive evolution of their ETDRS score. The model comparing the weight of the different variables on the change in ETDRS score over 24 months is represented in Fig. 1(and Additional file 1).

**Table 1:** Societal data for patients with a negative evolution of their ETDRS score over 24 months of follow-up (78.2% of patients): For negative ETDRS score evolution: 1 corresponds to changes between − 10 and − 5 letters; 2 corresponds to changes between − 5 and 0

Variable	Categories	Percentage
Negative ETDRS Score evolution	1	65.574
	2	34.426
Number of injections between baseline and M24	0	21.311
	1	13.115
	2	4.918
	3	13.115
	4	8.197
	5	6.557
	6	6.557
	7	6.557
	8	3.279
	9	1.639
	10	3.279
	12	3.279
	16	1.639
	17	1.639
	19	1.639
	20	1.639
	22	1.639
Outpatient follow-up	No	80.328
	Yes	16.393
	Sometimes	3.279
Family status	Couple	52.459
	Living alone	45.902
	Family	1.639
Transportation to the clinic	Medicalised vehicle	40.984
	Taxi	6.557
	Public transportation	3.279
	Personal car	47.541
	Walking	1.639
Commuting time to the clinic	0 to 1 h	42.623
	1 to 2 h	42.623
	More than 2 h	14.754
Low vision rehabilitation	No	88.525
	Yes	11.475
Daily reading	None	27.869
	Journal	29.508
	Books	32.787
	Computer	9.836
Health assurance covering 100% of expenses	No	34.426
	Yes	65.574
Economic burden of the treatment on the patient (0–10 scale)	1	4.918
	2	13.115
	3	19.672
	4	6.557
	5	24.590
	6	9.836
	7	18.033
	8	3.279
Painfulness of the treatment burden (0–10 scale)	1	8.197
	2	16.393
	3	29.508
	4	9.836
	5	21.311
	6	9.836
	7	1.639
	8	1.639
	10	1.639

**Table 2:** Societal data for patients with a positive evolution of their ETDRS score over 24 months of follow-up (21.8% of patients): for positive ETDRS score evolution (first line): 3 corresponds to changes between 0 and + 5 letters; 4 corresponds to changes between + 5 and + 10 letters

Variable	Categories	Percentage
Positive ETDRS Score evolution	3	70.588
	4	29.412
Number of injections between baseline and M24	0	5.882
	2	5.882
	5	17.647
	6	29.412
	8	5.882
	9	5.882
	10	5.882
	14	11.765
	15	5.882
	17	5.882
Outpatient follow-up	No	88.235
	Yes	5.882
	Sometimes	5.882
Family status	Couple	41.176
	Living alone	52.941
	Family	5.882
Transportation to the clinic	Medicalized vehicle	52.941
	Taxi	23.529
	Public transportation	5.882
	Personal car	17.647
Commuting time to the clinic	0 to 1 h	47.059
	1 to 2 h	47.059
	More than 2 h	5.882
Low vision rehabilitation	No	94.118
	Yes	5.882
Daily reading	None	11.765
	Journal	58.824
	Books	23.529
	Computer	5.882
Health assurance covering 100% of expenses	No	29.412
	Yes	70.588
Economic burden of the treatment on the patient (0–10 scale)	2	5.882
	3	17.647
	4	29.412
	5	17.647
	6	17.647
	7	5.882
	8	5.882
Painfulness of the treatment burden (0–10 scale)	2	17.647
	3	11.765
	4	23.529
	5	17.647
	6	5.882
	7	17.647
	8	5.882

**Fig. 2:**
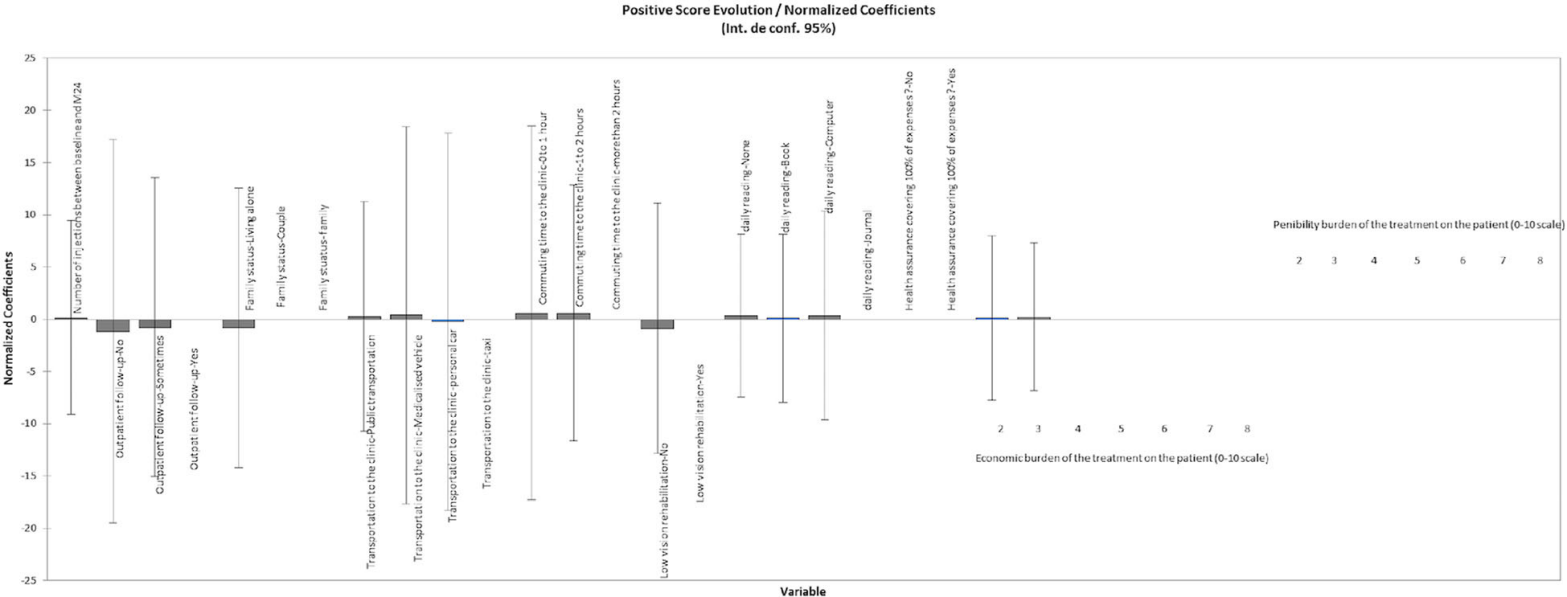
Graphical representation of the model assessing the weight (coefficient) of each variable on the change in ETRDS score over 24 months in the subgroup of nAMD patients who gained vision over 24 months

**Fig. 3:**
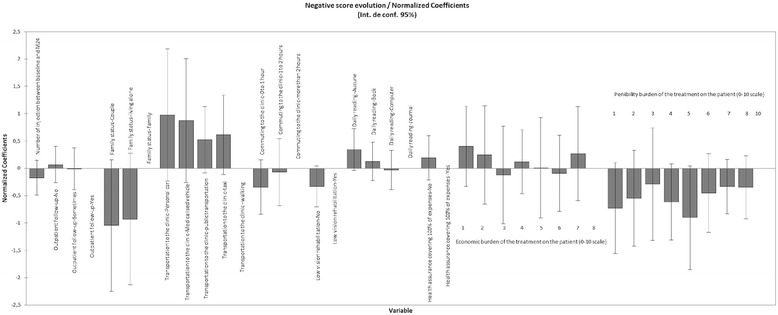
Graphical representation of the model assessing the weight (coefficient) of each variable on the change in ETRDS score over 24 months in the subgroup of nAMD patients who lost vision over 24 months

However, the ANCOVA analysis showed that 42.4% of the variability of the change in BCVA score between baseline and M24 was explained by the cumulative effect of all studied factors. The remaining variability was due to some other effects (other variables) that were not analysed in this study. Comparative effects of the weight of individual variables in the cohort of patients whose BCVA score improved (Fig. 2 and Additional file 1) and those whose BCVA score worsened (Fig. 3 and Additional file 1) at the end of the follow-up (M24) were studied.

Type I and Type III sum of square analysis showed that the treatment burdensome, low vision rehabilitation and daily reading variables provided the least information to the model (Fig. 1). It should be noted that the corresponding confidence interval included 0, confirming the relatively weak impact of each environmental factor taken separately on the change in BCVA. Regarding the clinical follow-up variable, it appeared that for a given injection number, the fact to be exclusively followed at the clinic was associated with a better change in BCVA than an intermittent outpatient follow-up. Living alone or in couple decreased the change in BCVA score over 24 months, compared to living with their family. Ambulance or taxi transportation contributed more to the increase in BCVA score than car or public transportation. We also found that a commuting time exceeding 2 h led to better effect than a shorter commuting time. Patients who used to read daily had a slight decrease in BCVA improvement compared to patients who did not read daily. Patients who paid their treatments themselves (no healthcare insurance) showed a trend to a higher BCVA gain than those who did not.

The main complaints of patients were the long waiting time before consultation (40% of patients), the cost of consultations and transport (30% of patients), the stress due to the injection and the unknown prognosis. Only one patient regretted having received this therapy because of these discomforts.

## Discussion

In our study, the ANCOVA analysis showed that 42.4% of the variability of the change in BCVA score from baseline could be explained by the cumulative effect of all studied factors. The remaining variability was due to other previously described environmental variables^7–16^ that were not analysed in this study. Because of the obvious evidence of the role of environmental factors in disease progression, our approach was to identify yet unknown societal factors that could influence nAMD evolution in patients treated with anti-VEGF therapy. These new factors appeared with the burden due to the need for monthly examination and/or treatment burden related to anti-VEGF therapy.

The large variability of factors influencing the healing process may explain why the variables assessed independently in our study did not provide a significant amount of information to the model. Since societal factors could have an impact on the healing process, population studies assessing these new societal criteria are needed [15]. Our study is, to the best of our knowledge, one of the first to investigate the impact of these new societal criteria, including intermittent outpatient follow-up, family status, type of transportation, commuting time, low vision rehabilitation, daily reading type, healthcare system, economic benefits of the social security scheme and the burdensome of the consultation on the nAMD patient treated with anti-VEGF therapy. Interestingly, patients with a large amount of painfulness due to the treatment (long commuting time, no healthcare insurance) seem to have better outcomes, probably because of a higher motivation and involvement in their treatment. Surprisingly, patients who reported having experienced pain related to the treatment also had a better BCVA gain than patients who did not. This is unexpected because it has been already reported that there is a relationship between pain and depression [16], between mood and sleep disorders and chronic pain [17], and finally that nAMD patients treated by antiVEGF experience a certain level of anxiety and depression [18]. It is also known that anxiety and depression could have a negative impact on the healing process of somatic diseases [19]. We can suggest in our study that the placebo effect in addition to the physiologic effect of antiVEGF therapy is higher when the injection is painful. Modulating a disease, which is partially determined by genetic risk factors, by societal factors is indeed possible and has already been shown in other diseases such as diabetes [20].

Investigating these parameters is essential because nAMD seems to be associated with depression or induce mood disorders [21] like numerous chronic diseases. All the factors analysed in this study could also lead to depression, including the family status or intermittent outpatient follow-up for instance. But depression or depressive mood could not only play a negative role on the healing process per se but also have a negative impact on the compliance with treatment, which could be detrimental to the functional outcomes at the end of the study. In this study, we eliminated the bias that these depression-related factors could introduce in terms of treatment discontinuation by excluding patients who did not complete the 2-year follow-up when they were exclusively followed in the clinic but it was not possible to exclude patients who were partially followed outside the clinic. Patient compliance could be one of the causes of lower BCVA gains in case of follow-up outside the clinic, but it could also be due to the longer access to treatment. Moreover, studies have shown that depression could maintain the disease process in chronic diseases [22–24]. Furthermore, it has been shown that neighbourhood deprivation is associated with age-related eye diseases [25], however, in nAMD, all the impacts of these factors remain to be elucidated and could be an important aspect to consider in patient treatment plan.

Another important aspect of these results is that the factors we studied could influence self-administered quality of life questionnaires used in nAMD studies. The assessment by patients themselves of their own quality of life could be biased by direct benefits from these factors, including the systematic use of an ambulance or the fact to be surrounded by their family at the time of the monthly consultations. Conversely, the self-assessment could be altered by the negative perception due to the heavy burden of the treatment. These elements should be kept in mind when interpreting quality of life scores in chronic diseases, especially in nAMD.

We might also wonder whether there might not be a bias in the studied population which is from the South of France where habits and family organization are different from those of the North of France and probably from other countries but are more representative of the family organization of Mediterranean countries, where a family circle is often present, the commuting time to the clinic is longer and the need for car use is frequent.

Finally, we can notice that the VA gain along the study is very lower than other studies such as the AURA study [26] for instance, − 7.5 letters in ours versus + 4.1 letters [26] at 2 years. We can assume that the reasons could be that our study includes patients a long time prior the AURA study (2010–2012) when the regimen of treatment in the real-life was less intensive than nowadays [27]; our baseline BCVA was slightly higher (58.35 versus 55 letters), and we did not include patients at the time of diagnosis but in October 2010 in order to assess the societal factors at the same period (October 2010–October 2012) for all patients, so we can have miss in our interval of follow-up the first months of treatment usually associated with the higher gain for patient. However the aim of our study was not to describe visual outcomes, but the impact of societal factors on maintaining visual gain over time in nAMD patients treated with antiVEGF.

## Conclusions

Societal factors seem to play an important yet unclear role in the course and functional outcomes in nAMD patients treated with anti-VEGFs. They may either contribute to the healing process or sustain the disease process together with stress or depression. Further studies will be needed to confirm our findings and identify all of these factors.

## Additional file

Additional file 1: Raw data: categories and frequencies of each different variable and model parameter values showing the coefficients of the different categories of each variable. (ODT 6 kb)

## Abbreviations

Anti-VEGF: Anti- vascular endothelial growth factor
BCVA: Best corrected visual acuity
DNA: Deoxyribonucleic acid
ETDRS: Early treatment diabetic retinopathy study
nAMD: Neovascular age-related macular degeneration
