# Supplementary material for: Influence of new societal factors on neovascular age-related macular degeneration outcomes
Source: BMC Ophthalmol. 2018 Feb 1;18:22. doi: 10.1186/s12886-018-0690-9 (PMC5793434; doi:10.1186/s12886-018-0690-9)
Supplement: Supplementary file 1 [file 12886_2018_690_MOESM1_ESM.odt]

Summary statistics:

| Variable | Categories | Frequencies | % |
| --- | --- | --- | --- |
| Evolution Score | -58 | 1 | 1,250 |
|  | -45 | 1 | 1,250 |
|  | -42 | 1 | 1,250 |
|  | -35 | 1 | 1,250 |
|  | -33 | 1 | 1,250 |
|  | -32 | 2 | 2,500 |
|  | -29 | 1 | 1,250 |
|  | -28 | 1 | 1,250 |
|  | -27 | 2 | 2,500 |
|  | -21 | 1 | 1,250 |
|  | -20 | 1 | 1,250 |
|  | -19 | 2 | 2,500 |
|  | -18 | 1 | 1,250 |
|  | -16 | 1 | 1,250 |
|  | -14 | 1 | 1,250 |
|  | -13 | 1 | 1,250 |
|  | -12 | 2 | 2,500 |
|  | -10 | 5 | 6,250 |
|  | -8 | 3 | 3,750 |
|  | -7 | 4 | 5,000 |
|  | -6 | 6 | 7,500 |
|  | -5 | 3 | 3,750 |
|  | -4 | 1 | 1,250 |
|  | -3 | 2 | 2,500 |
|  | -2 | 6 | 7,500 |
|  | -1 | 1 | 1,250 |
|  | 0 | 9 | 11,250 |
|  | 1 | 1 | 1,250 |
|  | 2 | 3 | 3,750 |
|  | 3 | 2 | 2,500 |
|  | 5 | 6 | 7,500 |
|  | 6 | 1 | 1,250 |
|  | 7 | 1 | 1,250 |
|  | 9 | 2 | 2,500 |
|  | 10 | 1 | 1,250 |
|  | 15 | 1 | 1,250 |
|  | 17 | 1 | 1,250 |
| Injection number since 2010 (baseline) | 1 | 1 | 1,250 |
|  | 2 | 3 | 3,750 |
|  | 3 | 7 | 8,750 |
|  | 4 | 4 | 5,000 |
|  | 5 | 7 | 8,750 |
|  | 6 | 10 | 12,500 |
|  | 7 | 6 | 7,500 |
|  | 8 | 3 | 3,750 |
|  | 9 | 5 | 6,250 |
|  | 10 | 5 | 6,250 |
|  | 11 | 5 | 6,250 |
|  | 12 | 9 | 11,250 |
|  | 13 | 3 | 3,750 |
|  | 14 | 3 | 3,750 |
|  | 15 | 1 | 1,250 |
|  | 17 | 1 | 1,250 |
|  | 18 | 4 | 5,000 |
|  | 19 | 2 | 2,500 |
|  | 21 | 1 | 1,250 |
| Injection number since 2012 | 2 | 2 | 2,500 |
|  | 3 | 4 | 5,000 |
|  | 5 | 6 | 7,500 |
|  | 6 | 3 | 3,750 |
|  | 7 | 8 | 10,000 |
|  | 8 | 4 | 5,000 |
|  | 9 | 3 | 3,750 |
|  | 10 | 2 | 2,500 |
|  | 11 | 4 | 5,000 |
|  | 12 | 5 | 6,250 |
|  | 13 | 1 | 1,250 |
|  | 14 | 2 | 2,500 |
|  | 15 | 1 | 1,250 |
|  | 16 | 7 | 8,750 |
|  | 17 | 2 | 2,500 |
|  | 18 | 4 | 5,000 |
|  | 19 | 3 | 3,750 |
|  | 20 | 2 | 2,500 |
|  | 21 | 3 | 3,750 |
|  | 24 | 3 | 3,750 |
|  | 27 | 1 | 1,250 |
|  | 28 | 2 | 2,500 |
|  | 29 | 4 | 5,000 |
|  | 32 | 1 | 1,250 |
|  | 34 | 1 | 1,250 |
|  | 37 | 1 | 1,250 |
|  | 41 | 1 | 1,250 |
| Injection number between  2010 and 2012 | 0 | 14 | 17,500 |
|  | 1 | 8 | 10,000 |
|  | 2 | 4 | 5,000 |
|  | 3 | 8 | 10,000 |
|  | 4 | 5 | 6,250 |
|  | 5 | 7 | 8,750 |
|  | 6 | 9 | 11,250 |
|  | 7 | 5 | 6,250 |
|  | 8 | 3 | 3,750 |
|  | 9 | 2 | 2,500 |
|  | 10 | 3 | 3,750 |
|  | 11 | 1 | 1,250 |
|  | 12 | 2 | 2,500 |
|  | 14 | 2 | 2,500 |
|  | 15 | 1 | 1,250 |
|  | 16 | 1 | 1,250 |
|  | 17 | 2 | 2,500 |
|  | 19 | 1 | 1,250 |
|  | 20 | 1 | 1,250 |
|  | 22 | 1 | 1,250 |
| Out clinic follow-up | No | 66 | 82,500 |
|  | Yes | 11 | 13,750 |
|  | Sometimes | 3 | 3,750 |
| Vie 1 | Couple | 40 | 50,000 |
|  | Alone | 37 | 46,250 |
|  | caregiver; family | 3 | 3,750 |
| Type of transportation Home - Clinic | Ambulance | 36 | 45,000 |
|  | taxi | 8 | 10,000 |
|  | Public transportation | 3 | 3,750 |
|  | Personal car | 32 | 40,000 |
|  | Walk | 1 | 1,250 |
| Transportation duration Home - Clinic | < 1 hours | 34 | 42,500 |
|  | 1 to 2 hours | 36 | 45,000 |
|  | > 2 hours | 10 | 12,500 |
| Low vision rehabilitation ? | No | 72 | 90,000 |
|  | Yes | 8 | 10,000 |
| Daily reading | None | 19 | 23,750 |
|  | Newspaper | 29 | 36,250 |
|  | Books | 25 | 31,250 |
|  | Computer | 7 | 8,750 |
| Healthcare reimbursement ? | No | 26 | 32,500 |
|  | Yes | 54 | 67,500 |
| Economic burden on the patient | 1 | 3 | 3,750 |
|  | 2 | 9 | 11,250 |
|  | 3 | 15 | 18,750 |
|  | 4 | 9 | 11,250 |
|  | 5 | 19 | 23,750 |
|  | 6 | 9 | 11,250 |
|  | 7 | 12 | 15,000 |
|  | 8 | 4 | 5,000 |
|  | 1 | 5 | 6,250 |
|  | 2 | 13 | 16,250 |
|  | 3 | 21 | 26,250 |
|  | 4 | 10 | 12,500 |
|  | 5 | 17 | 21,250 |
|  | 6 | 7 | 8,750 |
|  | 7 | 4 | 5,000 |
|  | 8 | 2 | 2,500 |
|  | 10 | 1 | 1,250 |

Table 1 : Summary statistics showing frequencies and percentages of each category of the different variables.

| Source | Value | Standard error | t | Pr > │t│ | Lower bound (95%) | Upper bound (95%) |
| --- | --- | --- | --- | --- | --- | --- |
| Injection number 2010 and 2012 | 0.089 | 0.129 | 0.691 | 0.493 | -0.171 | 0.350 |
| Out clinic follow—up-No | 0.218 | 0.268 | 0.813 | 0.420 | -0.321 | 0.756 |
| Out clinic follow—up-Yes | -0.006 | 0.269 | -0.021 | 0.983 | -0.546 | 0.535 |
| Out clinic follow—up-Sometimes | 0.000 | 0.000 |  |  |  |  |
| Living-Couple | -0.739 | 0.400 | -1.850 | 0.071 | -1.543 | 0.064 |
| Living-Alone | -0.480 | 0.389 | -1.236 | 0.223 | -1.262 | 0.302 |
| Living-Help from caregiver or family | 0.000 | 0.000 |  |  |  |  |
| Transportation - Ambulance | 0.611 | 0.589 | 1.036 | 0.305 | -0.575 | 1.796 |
| Transportation - taxi | 0.414 | 0.377 | 1.100 | 0.277 | -0.344 | 1.172 |
| Transportation- public transportation | 0.152 | 0.256 | 0.594 | 0.555 | -0.363 | 0.668 |
| Transportation - Personal car | 0.268 | 0.585 | 0.459 | 0.649 | -0.908 | 1.444 |
| Transportation - walk | 0.000 | 0.000 |  |  |  |  |
| Transportation duration-0 to 1 hour | -0.144 | 0.227 | -0.633 | 0.530 | -0.600 | 0.313 |
| Transportation duration - 1 to 2 hours | -0.046 | 0.243 | -0.190 | 0.850 | -0.534 | 0.442 |
| Transportation duration-more than 2 hours | 0.000 | 0.000 |  |  |  |  |
| Low vision rehabilitation ?-No | -0.015 | 0.157 | -0.095 | 0.925 | -0.330 | 0.301 |
| Low vision rehabilitation ?-Yes | 0.000 | 0.000 |  |  |  |  |
| Daily reading-None | 0.095 | 0.217 | 0.437 | 0.664 | -0.342 | 0.532 |
| Daily reading- Newspaper | -0.044 | 0.245 | -0.181 | 0.858 | -0.536 | 0.448 |
| Daily reading-Books | -0.023 | 0.237 | -0.098 | 0.922 | -0.501 | 0.454 |
| Daily reading-Computer | 0.000 | 0.000 |  |  |  |  |
| Healthcare reimbursement ?-No | 0.220 | 0.169 | 1.299 | 0.200 | -0.121 | 0.560 |
| Healthcare reimbursement ?-Yes | 0.000 | 0.000 |  |  |  |  |
| Economic burden-1 | 0.039 | 0.263 | 0.148 | 0.883 | -0.490 | 0.568 |
| Economic burden-2 | -0.227 | 0.259 | -0.874 | 0.387 | -0.748 | 0.295 |
| Economic burden-3 | -0.078 | 0.277 | -0.283 | 0.778 | -0.635 | 0.478 |
| Economic burden-4 | -0.107 | 0.233 | -0.462 | 0.646 | -0.575 | 0.360 |
| Economic burden-5 | -0.182 | 0.273 | -0.667 | 0.508 | -0.731 | 0.367 |
| Economic burden-6 | -0.063 | 0.233 | -0.271 | 0.787 | -0.531 | 0.405 |
| Economic burden-7 | -0.105 | 0.269 | -0.392 | 0.697 | -0.646 | 0.435 |
| Economic burden-8 | 0.000 | 0.000 |  |  |  |  |
| Personal burden-1 | -0.250 | 0.373 | -0.671 | 0.506 | -1.001 | 0.500 |
| Personal burden-2 | -0.507 | 0.445 | -1.140 | 0.260 | -1.401 | 0.388 |
| Personal burden-3 | -0.359 | 0.524 | -0.684 | 0.497 | -1.413 | 0.696 |
| Personal burden-4 | -0.204 | 0.390 | -0.522 | 0.604 | -0.988 | 0.581 |
| Personal burden-5 | -0.693 | 0.493 | -1.405 | 0.167 | -1.685 | 0.299 |
| Personal burden-6 | -0.184 | 0.340 | -0.540 | 0.592 | -0.868 | 0.501 |
| Personal burden-7 | -0.114 | 0.276 | -0.415 | 0.680 | -0.669 | 0.440 |
| Personal burden-8 | 0.026 | 0.243 | 0.106 | 0.916 | -0.464 | 0.515 |
| Personal burden-10 | 0.000 | 0.000 |  |  |  |  |

Table 2 : Model parameters values showing the coefficients of the different categories of each variable.
